# Evaluation of Children with Chronic Rhinosinusitis after Adenotonsillectomy

**Published:** 2012

**Authors:** Fatholah Behnoud

**Affiliations:** Associate Professor Hamedan university of medical sciences ENT department Besat Hospital

**Keywords:** Adenoid, Adenoidectomy, Palatine tonsil, Tonsillectomy

## Abstract

**Introduction:**

Chronic rhinosinusitis (CRS), defined as an inflammatory process involving the paranasal sinuses that continues for at least three months, is a major cause of morbidity in the pediatric population and a difficult entity to treat with a poorly defined pathophysiology. The cornerstone of treatment for children with CRS remains aggressive antibiotic therapy, but many patients fail to improve even after extended courses of broad-spectrum oral antibiotics. However, good treatment results with adenoidectomy alone have been reported in pediatric patients with CRS. The purpose of this study was to evaluate the effect of adenotonsillectomy on chronic rhinosinusitis in children.

**Materials and Methods:**

In this clinical trial the study population was 40 children under 14 years old who had been selected for adenotonsillectomy. Prior to the procedure, a Waters’ view radiograph was performed on individuals that suffered from CRS and displayed symptoms such as rhinorrhea, halitosis, and chronic cough. Only patients with bilateral clouding of the maxillary sinuses were enrolled in study. A further radiograph was performed on the 28^th^ day following the procedure and the outcome of the treatment evaluated.

**Results:**

Of the 40 patients under 14 years old who were evaluated, 22 (55%) were female and 18 (45%) were male. The mean age of the patients was 7.22 years while the oldest was 14 and the youngest was 4 years old. Nasal congestion, rhinorrhea, post nasal drip, and chronic cough were present in all of the patients. Following the adenotonsillectomy, these symptoms were significantly reduced and were present in only 15.5%, 0%, 20%, and 20% of the patients, respectively. Multivariate analyses were performed using McNemar’s test.

**Conclusion:**

According to the results of this study, where 72.5% of patients showed a complete recovery following treatment, an adenotonsillectomy can be considered as a treatment modality for CRS.

## Introduction

Recurrent and chronic infections of the tonsils and adenoids continue to be a common problem, and adenotonsillectomy remains a commonly performed surgical procedure (1, 2). Rhinosinusitis is a common diagnosis in children and has been shown to significantly impact patients and their families (3). Chronic rhinosinusitis (CRS) is diagnosed when a child experiences symptoms of nasal congestion, rhinorrhea, post nasal drip, halitosis, cough, headaches, or fevers secondary to inflammation of the nose and paranasal sinuses that persist for longer than three months (4-6). The cornerstone of treatment for children with CRS remains aggressive antibiotic therapy, but many patients fail to improve even after extended courses of broad-spectrum oral antibiotics (5, 7). 

Alternative treatments include functional endoscopic sinus surgery (FESS), which has been shown to be effective in these patients, but concerns exist regarding its effects on the development of the facial skeletal (5). Good treatment results have also been reported in pediatric CRS following adenoidectomy (6) and the use of a regimen consisting of intravenous antibiotic therapy with a concurrent sinus aspiration has been shown to symptomatically ameliorate CRS in pediatric patients who failed to respond to conventional oral therapy (7).

Because biofilms have been implicated as a nidus for chronic bacterial infection in children with CRS (8) and the histology of the adenoid and tonsils are similar and both are located in Waldeyer’s ring, we describe our recent experience in managing a series of pediatric patients with CRS who underwent adenotonsillectomy instead of adenoidectomy alone. The purpose of this study was to evaluate the effect of adenotonsillectomy on CRS in children.

## Materials and Methods

This cross-sectional study evaluated 40 children younger than 14 years old (18 males and 22 females) from a larger sample population who were consecutively referred to the Ear, Nose and Throat (ENT) Department of Besat Hospital in Hamedan, between March 2009 and March 2010, for adenotonsillectomy due to a variety of indications including recurrent acute tonsillitis, adenoid hypertrophy associated with chronic sinusitis, chronic mouth-breathing, sleep disturbances, and chronic otitis media with effusion. To evaluate the patients’ adenotonsillar status we performed a lateral nasopharyngeal radiograph in order to determine the size of the adenoid and directly inspect the tonsils according to the Brodsky Scale. The 40 patients enrolled in the study were selected from the total number of candidates based on the clinical criteria of the presence of CRS (symptoms of which included purulent rhinorrhea, post nasal drip, chronic cough, halitosis, and so on) that had persisted for longer than 3 months and had not responded to routine oral antibiotic therapy. All of the 40 patients also had to have bilateral mucosal thickening of the maxillary sinuses as detected by a Water’s view x-ray.

On admission, a detailed clinical history was obtained for each patient and a thorough physical examination was carried out by an otolaryngologist. All the children underwent routine adenotonsillectomy and for 2 weeks after the surgery they had taken amoxicillin 50 mg/kg/day and for 3 days acetaminophen, 15 mg/kg/6 hours. All patients were discharged the day after surgery and followed the same oral antibiotic and analgesic regimen postoperatively. Children with systemic disease or other abnormalities were excluded. Additionally, those with fungal sinusitis or previous surgery on the sinuses or polyposis were excluded.

All patients were followed after surgery and visited by an otolaryngologist at 7, 14, and 28 days following surgery. On the 28^th^ day, Water’s view radiography was performed again and the degree of improvement was rated according to the following scores: 1, unilateral maxillary sinus was clear; 2, bilateral sinuses show improvement over preoperative radiography; 3, no change was seen in the radiograph; 4, bilateral sinuses were completely clear. A score of 1 or 2 indicated a partial improvement, 3 indicated no improvement, and 4 indicated a complete recovery.

Clinical findings were matched with the Water’s view radiograph and a questionnaire was used to assess the status of the major and minor symptoms. The questionnaire was administered by a nurse to the parents of the patient, who ranked each preoperative symptom as cured, better, same, or worse.

The parents were informed about the study protocols and informed consent was obtained and the study was approved by our Ethical Committee. Comparison of the study variables before and after the intervention was performed using the related groups test (McNemar’s test) with P< 0.05 indicating statistical significance.

## Results

Of the 40 patients under 14 years old who were evaluated in this study 22 (55%) were female and 18 (45%) were male. The mean age of the patients was 7.22 years with the oldest being 14 and the youngest 4 years old (Table 1).

**Table 1:** Baseline characteristics of the patients

Patient Characteristic	Female N = 22 (55%)	Male N = 18 (45%)	Total N = 40 (100%)
Age (years, mean ± SD)Range	8.18 ± 2.74 4–14	6.1 ± 2.44 4–14	7.22 ± 2.78 4–14
Weight (kg, mean ± SD)Range	26.27 ± 7.89 15–47	21.55 ± 6.99 15–42	24.15 ± 7.78 15–47

Preoperatively, all 40 (100%) of the patients suffered from nasal congestion, purulent rhinorrhea, post nasal drip, and cough. On the 28^th^ day following surgery these symptoms were present in 15.5%, 0%, 20%, and 20% of patients, respectively (P = 0.000 for all symptoms). Halitosis was seen in 39 (97.5%) of the patients preoperatively but was only seen in 8 (20%) patients in the post-operative period. 

 Preoperatively, 20 (50%) of the patients suffered from headaches in comparison with 1 (2.5%) who suffered from headache and 29 (72.5%) who had a complete recovery postoperatively (Table 2). With regards to the radiological findings at 28 days following adenotonsillectomy, 29 (72.5%) patients showed complete clearing of the mucosal thickening of the maxillary sinuses and 11 (27.5%) showed partial changes.

**Table 2:** Frequency distribution of symptoms of CRS before and after adenotonsillectomy

Symptoms and Signs	Before Adenotonsillectomy N (%)	After Adenotonsillectomy N (%)	Change	P *
Nasal congestion	40 (100)	7 (17.5)	82.5%	0.000
Rhinorrhea	40 (100)	0 (0)	100%	0.000
PND	40 (100)	8 (20)	80%	0.000
Halitosis	39 (97.5)	8 (20)	77.5%	0.000
Cough	40 (100)	8 (20)	80%	0.000
Headache	20 (50)	1 (2.5)	47.5%	0.000

* According to McNemar’s test; PND, post nasal discharge

## Discussion

CRS remains a significant problem for physicians dealing with the pediatric population. This study shows the effectiveness of adenotonsillectomy in the management of children with CRS and adds to the data provided by several prospective outcome studies that have shown improvements in disease-specific and global quality of life after adenotonsillectomy (2, 9).

The precise diagnosis of CRS in children is often difficult to make because of the overlap of symptomatology with more common conditions such as viral upper respiratory infections and adenoid infection or hypertrophy (10, 11). Recent findings suggest that adenoidectomy by itself may provide benefits for patients with CRS. In a study by Vandenberg and Healthy, 58% of children demonstrated near or complete symptom resolution of CRS after adenoidectomy (10, 12). Scott and colleagues in their meta-analysis of the effect of adenoidectomy on CRS estimated that approximately 70% of patients benefit symptomatically from adenoidectomy alone (9). Support for the benefits of adenoidectomy has also come from studies that show a reduction in the number of bacterial pathogens and an increase in commensal microorganisms in the nasopharynx after adenoidectomy (13). In addition, there have been several studies on the effect of tonsillectomy for reduction of episodes of pharyngitis (1) and clearing of the pharynx of pathogenic microorganisms. Therefore, the size of the adenoid and the presence of associated diseases are factors for consideration (14) and our experience is that tonsillectomy added to adenoidectomy can improve the treatment process for CRS.

In our study, nasal congestion, purulent rhinorrhea, post nasal drip, and cough were seen in all 40 (100%) patients preoperatively but 4 weeks after adenotosillectomy the symptoms had disappeared in the majority of patients. Postoperatively 7 (15.5%) had nasal congestion, 0 (0%) patients had purulent rhinorrhea, 8 (20%) had post nasal drip, and 8 (20%) had a cough. In this study, halitosis was seen in 39 (97.5%) patients before surgery and only in 8 (20%) patients after adenotonsillectomy. Headaches were reported by 20 (50%) patients before intervention and only 1 (2.5%) patient suffered from them afterwards.

Further evidence for the usefulness of surgical intervention in pediatric CRS comes from various studies including one in China, which evaluated the effect of two different treatments for CRS. Long-term, low-dose macrolides were shown to be an effective therapy and a valid alternative in pediatric CRS. Surgical intervention was necessary for cases that did not respond to the prolonged course of medical treatment. Adenoidectomy and/or tonsillectomy was the recommended surgical procedure for children with adenoid and/or tonsil hypertrophy (15). Brietzke and Brigger, in a meta-analysis of the outcomes of adenoidectomy on CRS, documented that adenoidectomy reduces caregiver-reported symptoms of CRS in the majority of pediatric patients. Given its simplicity, low risk profile, and apparent effectiveness, adenoidectomy should be considered first-line therapy for medically refractory, uncomplicated pediatric rhinosinusitis (16). Ramadan compared the efficacy of FESS and adenoidectomy with FESS alone and showed that certain children did better when FESS was performed in conjunction with adenoidectomy in comparison with FESS alone (13). Price and colleagues, in their study of the efficacy of adenoidectomy in children with Down syndrome, indicated that children undergoing both adenoidectomy and tonsillectomy showed a greater improvement in snoring and apnea than those undergoing adenoidectomy alone. The study did not discuss sinonasal status (17).

In this study, a significant improvement in the symptoms of CRS in a pediatric group was obtained following adenotonsillectomy. The clinical findings improved in nearly all of the patients and bilateral mucosal thickening of the maxillary sinuses, as detected by Water’s view radiography, disappeared in 72.5% of patients. The author believes that the positive results of this study are related to the concept that completely removing the infectious complex (adenotonsils) is superior to the administration of antibiotics.

## Conclusion

The results of this study suggest that adenotonsillectomy improves the treatment process of pediatric CRS.
